# Fibroepithelial Lesion Initially Believed to Be Fibroadenoma, but Interval Growth Consistent With Phyllodes Tumor

**DOI:** 10.7759/cureus.10363

**Published:** 2020-09-10

**Authors:** Quan D Nguyen, Samuel O Krider, James T Roberts, Flavia E Posleman Monetto, Jing He

**Affiliations:** 1 Radiology, University of Texas Medical Branch, Galveston, USA; 2 Diagnostic Radiology, University of Texas Medical Branch, Galveston, USA; 3 Pathology, University of Texas Medical Branch, Galveston, USA

**Keywords:** fibroadenoma, phyllodes, fibroepithelial, mammogram, breast mass, breast cancer, ultrasound, breast cancer screening

## Abstract

Fibroepithelial lesions of the breast are commonly encountered tumors comprised of stromal and epithelial components. Fibroadenoma and phyllodes tumor are both fibroepithelial lesions, but their management differs. Phyllodes tumor requires surgical excision, whereas fibroadenoma requires no further workup. Both have many overlapping histological features making it difficult to distinguish between a benign fibroadenoma versus the more aggressive phyllodes tumor. This case details a breast mass that was initially believed to be a fibroadenoma, but interval growth at one year follow up resulted in surgical excision with final pathology revealing phyllodes tumor.

## Introduction

Fibroepithelial lesions of the breast occur in approximately 10-15% of women [1]. Phyllodes tumors of the breast account for 0.3% to 1% of all primary breast tumors and constitute 2.5% of all fibroepithelial tumors [2, 3]. Phyllodes tumors are graded according to recommendations by the World Health Organization as benign, borderline, or malignant based on the presence of stromal cellularity, atypia, mitotic activity, border infiltrations versus circumscription, and stromal overgrowth [4-6]. While benign and borderline phyllodes tumors demonstrate a good prognosis and low rates of recurrence, malignant phyllodes tumors often metastasize, exhibit poor clinical prognosis, and demonstrate high rates of recurrence. Given such clinical variability, accurate grading, and differentiation of phyllodes tumors are imperative. Grading requires evaluation of the entire lesion and should be based on excision specimens [6-8].

## Case presentation

**Figure 1:**
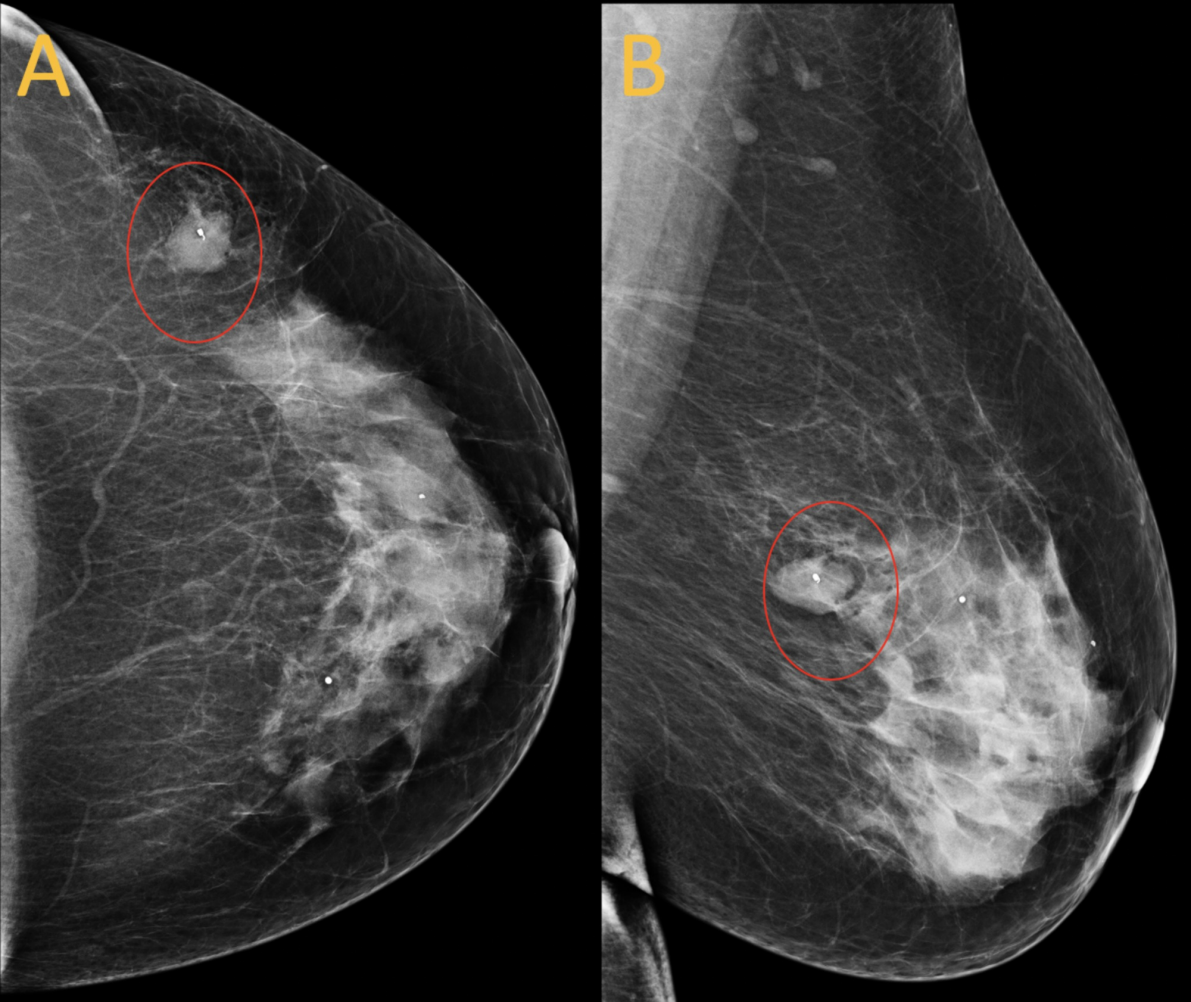
Initial post-biopsy mammogram Images show left breast oval mass with circumscribed margins measuring 15 mm (red circles). A mammogram was performed in January 2019.

**Figure 2:**
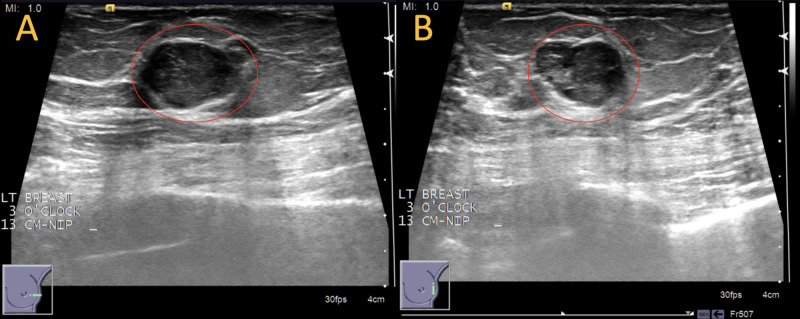
Left breast diagnostic ultrasound Images show left breast oval mass with circumscribed margins measuring 15 mm (red circles). An ultrasound was performed in January 2019.

**Figure 3:**
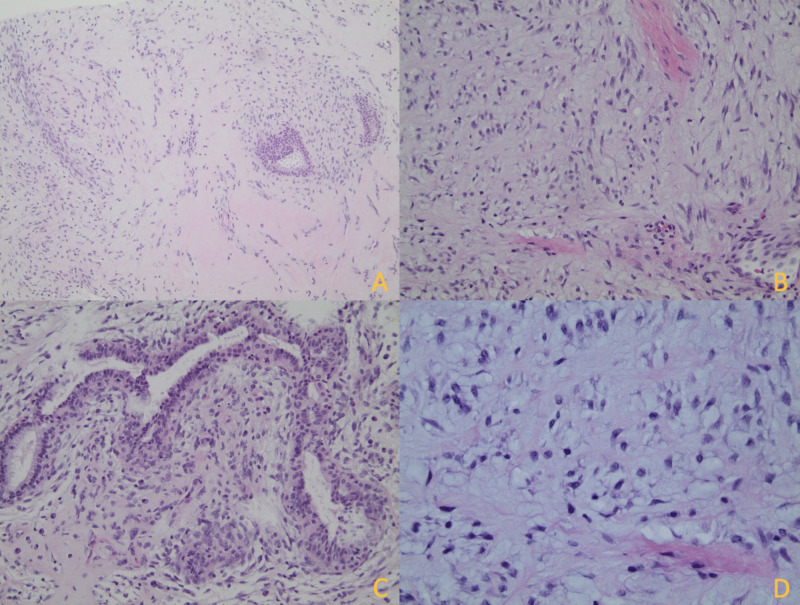
Histological findings of biopsy of the breast mass A: An ultrasound-guided core biopsy of the breast mass shows a fibroepithelial lesion with alternating hypercellular and hypocellular stroma (magnification x100). B: Stromal cells are organized in fascicles (magnification x200). C: Increased cellularity (condensation) is seen adjacent to the epithelium (magnification x200). D: Stromal cells show cytological atypia with nuclear pleomorphism and hyperchromatic nuclei (magnification x400).

A 64-year-old woman with a past medical history of type 2 diabetes mellitus and hypertension presented for her annual bilateral screening mammogram. The patient reported no relevant family, medical, or surgical history. Screening mammogram images of the left breast demonstrated a 17 mm oval-shaped mass with circumscribed margins at the three o’clock position in posterior depth, 13 cm from the nipple (Figure 1). Targeted ultrasound of the right breast demonstrated an oval-shaped hypoechoic circumscribed mass measuring 15 x 9 x 12 mm at three o'clock, 13 cm from the nipple with the orientation that is parallel to the skin (Figure 2). An ultrasound-guided core biopsy of the breast mass was performed with pathology revealing a fibroepithelial lesion favoring fibroadenoma, although phyllodes tumor could not be ruled out (Figure 3).

**Figure 4:**
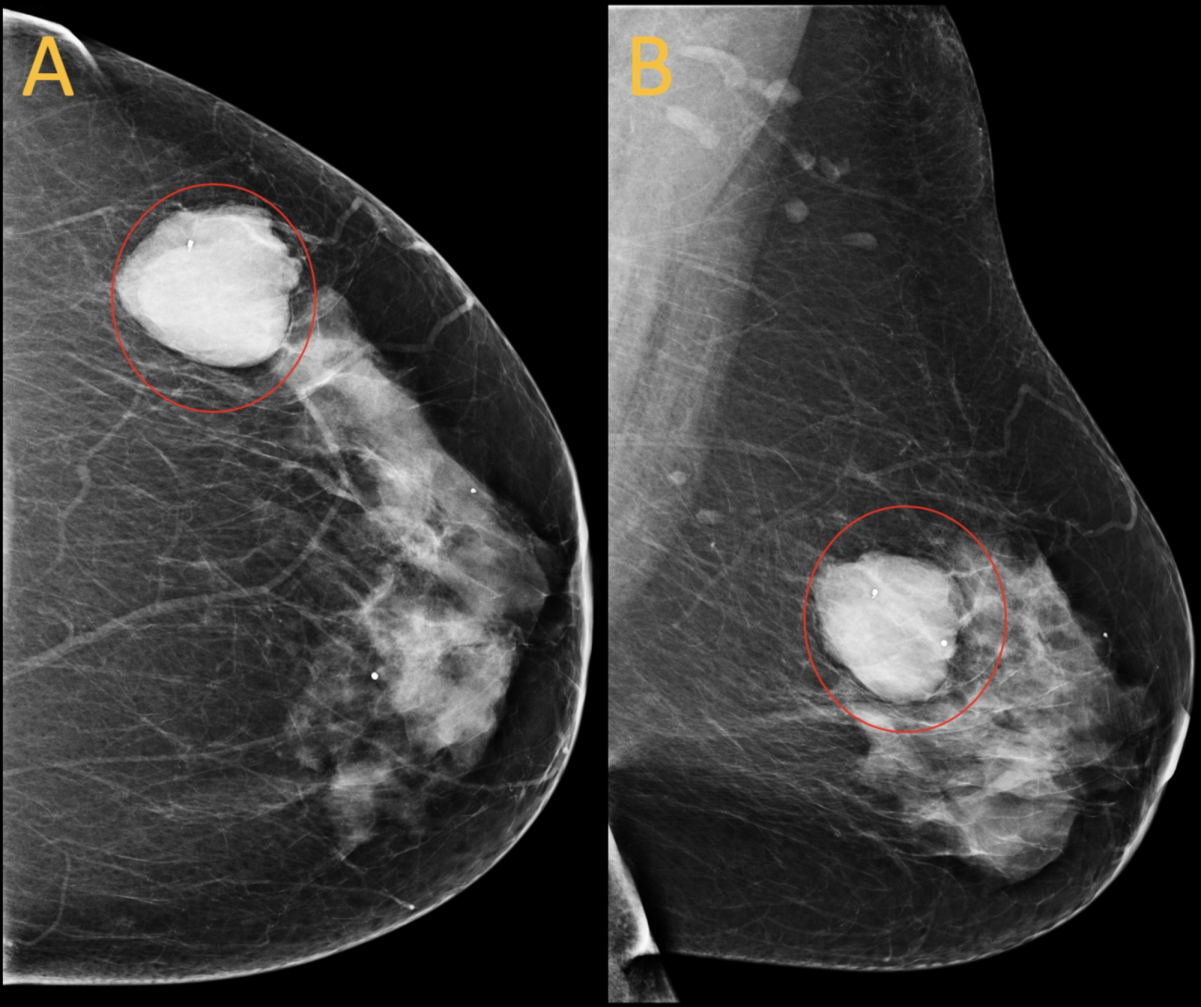
Screening mammogram one year later A 46 mm mass containing a biopsy clip is demonstrated at three o'clock, 13 cm from the nipple (red circles). The mass increased from 15 mm on the prior mammogram the year before. A mammogram was performed in January 2020.

**Figure 5:**
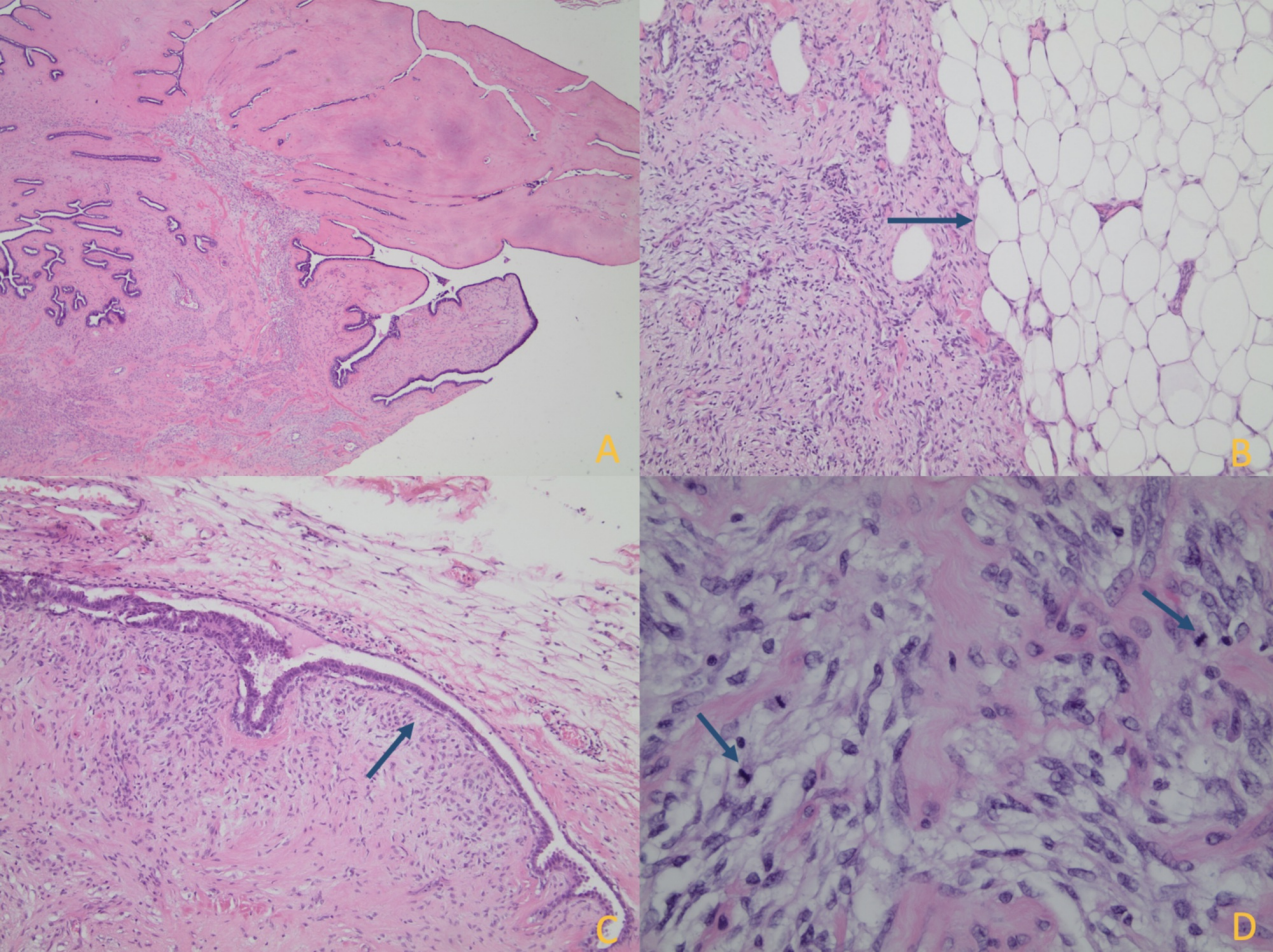
Histological findings of lumpectomy of the borderline phyllodes tumor A: H&E staining of the mass shows lead-like fronds (magnification x20). B: Tumor invades into the adipose tissue (magnification x100). C: Increased stromal cellularity adjacent to the epithelium (magnification x100). D: Stromal cells show pleomorphic nuclei and mitotic figures (magnification x400).

Follow up screening mammogram one year after ultrasound guided core needle biopsy showed a 46 mm mass with an associated clip marker at three o’clock, 13 cm from the nipple (Figure 4). This mass increased in size when compared with initial screening mammogram a year ago when it measured 15 mm. Given marked interval increase in size of tumor along with biopsy proven fibroepithelial lesion, concern for malignant phyllodes tumor was raised and surgical excision was recommended. Patient underwent surgical consultation followed by lumpectomy which demonstrated borderline phyllodes tumor, intermediate grade (Figure 5).

## Discussion

The vast majority of phyllodes tumors occur in women, with a median presenting age of 42 to 45 years (range 10 to 82 years). A fibroadenoma is the most common solid breast mass in a woman under the age of 30 but can be seen in older women as well.

The factors that significantly helped to identify phyllodes tumors upon univariable analysis consisted of the presenting symptoms (palpable mass or breast pain), increased size on clinical examination, hyperdense mass on the mammogram, and the following three ultrasound features: heterogeneous echogenicity, presence of round cysts within the mass, and presence of clefts within the mass. The pathologist's suggestion of a phyllodes tumor is also helpful. The symptoms of breast pain, the presence of clefts on ultrasound, the presence of round cysts on ultrasound, and the pathologist favoring of phyllodes tumors from a core needle biopsy specimen are factors that remained statistically significant upon multivariable analysis [9].

Ultrasound findings (clefts and round cysts), suggestive pathological diagnoses, and clinical symptoms are all useful for the decision to surgically remove fibroepithelial lesions diagnosed from the core needle biopsy.

## Conclusions

This case shows the challenge of distinguishing a fibroadenoma from phyllodes tumor on both breast imaging and tissue analysis. In cases where the pathologist reports fibroepithelial lesion and cannot definitively call fibroadenoma on a core needle biopsy sample, a radiologist can help diagnose phyllodes tumor earlier by recommending surgical excision immediately for patients over 40 years old or by recommending short interval follow up imaging in six months to detect interval growth.
